# Activity Expression and Property Analysis of Codon-Optimized Polyphenol Oxidase from *Camellia sinensis* in *Pichia pastoris* KM71

**DOI:** 10.3390/foods14152749

**Published:** 2025-08-06

**Authors:** Xin Zhang, Yong-Quan Xu, Jun-Feng Yin, Chun Zou

**Affiliations:** Tea Research Institute, Chinese Academy of Agricultural Sciences, Key Laboratory of Biology, Ministry of Agriculture and Rural Affairs, 9 South Meiling Road, Hangzhou 310008, China; xinzhang@tricaas.com (X.Z.); yqx33@126.com (Y.-Q.X.); yinjf@tricaas.com (J.-F.Y.)

**Keywords:** *Camellia sinensis*, polyphenol oxidase, *Pichia pastoris*, codon optimization, enzymatic properties

## Abstract

Tea polyphenol oxidase (CsPPO) is a crucial enzyme involved in the production of tea and tea products. However, the recombinant expression of CsPPO in microorganisms is often hindered by challenges such as inclusion body formation and extremely low enzyme activity. In this study, the *CsPPO* gene (1800 bp) from *Camellia sinensis* cv. Yihongzao was cloned and 14.5% of its codons were optimized for *Pichia pastoris* expression. Compared to pre-optimization, codon optimization significantly enhanced CsPPO production in *P. pastoris* KM71, yielding a 42.89-fold increase in enzyme activity (1286.67 U/mL). The optimal temperature and pH for recombinant CsPPO were determined to be 40 °C and 5.5, respectively. This study demonstrates that codon optimization effectively improves the expression of plant-derived enzymes such as CsPPO in eukaryotic expression systems. Future research should explore the long-term stability of recombinant CsPPO and its potential applications in tea fermentation processes and functional food development.

## 1. Introduction

Polyphenol oxidase (PPO), a member of the oxidoreductases family, is widely distributed in plants [1], animals [2], and fungi [3]. In tea derived from the leaves of the tea plant (*Camellia sinensis* (L.) O. Ktze), polyphenol oxidase (PPO) exerts a crucial influence on the oxidation degree during processing, thereby significantly impacting the quality of products such as white, oolong, and black teas [4]. CsPPO has also been applied in the preparation of catechin oxidation products [5], including theaflavins and theasinensins. Moreover, CsPPO is also involved in defending against pathogens and pests, playing a role in innate and adaptive immunity throughout all growth stages of the tea plant [6]. Due to the important role of CsPPO in the tea industry, researchers are striving to achieve its low-cost and large-scale preparation [7].

Methods for CsPPO extraction from fresh tea leaves include acetone extraction [8], buffer extraction [9], and surfactant extraction [10]. However, these methods are limited by low activity, complicated processes, seasonal variability, and transportation, making them difficult to apply to large-scale preparation [11]. Therefore, extensive research has been conducted on obtaining CsPPO through microbial recombinant expression.

The prokaryotic expression system is widely used for the recombinant expression of CsPPO, but its expression has been largely unsuccessful. Four *CsPPO* genes from *C. sinensis* var. assamica were cloned into *Escherichia coli*, all of which were expressed in the form of inclusion bodies [12]. In order to express active CsPPO through *E. coli*, the expression vectors pET32a and pMALc5X were used [13]. However, the protein expressed in the pET32a vector existed in the insoluble form, while CsPPO expressed in the pMAL-c5X vector, although soluble, had low activity. Other studies [14,15] also indicate that CsPPO expression in *E. coli* easily forms inclusion bodies, possibly due to the difficulty of the active expression of genes from eukaryotic organisms in prokaryotic expression systems.

*Pichia pastoris* is a commonly used eukaryotic expression system that has also been used for the recombinant expression of CsPPO. The secretory and non-secretory vectors pPICZαA and pPICZA were used for the expression of CsPPO in *P. pastoris* GS115 [16], but both had low expression levels. In addition to the expression system, there are other factors that can affect the expression level, among which codon preference is a crucial factor. For example, the expression level of the berberine bridge enzyme gene was increased by 58 times through codon optimization [17]. To achieve the high-activity expression of CsPPO, this study synonymously replaced rare codons in its encoded gene and expressed them in *P. pastoris* KM71, for which a patent was applied (application number: CN 202311760710.X) in China. In addition, the enzymatic properties of the recombinant enzyme were analyzed. This study aimed to address these limitations through the systematic codon optimization and comprehensive characterization of recombinant CsPPO, thereby providing a valuable reference for the large-scale preparation of CsPPO.

## 2. Materials and Methods

### 2.1. Reagents and Materials

The first expanding leaves of *C. sinensis* var. Yihongzao were sampled from the National Tea Germplasm Repository Hangzhou (NTGRH), China. The yeast expression vector pPIC3.5K and YPD medium were purchased from Sangon Biotech Co., Ltd. (Shanghai, China). The *P. pastoris* KM71 strain was purchased from Coolaber Co., Ltd. (Beijing, China). Proline, NaCl, Na_2_HPO_4_, citric acid, and other reagents were purchased from Sinopharm Chemical Reagent Co., Ltd. (Shanghai, China).

### 2.2. Genomic DNA Extraction

The genomic DNA of the tea plant was extracted with the Plant Genome Extraction Kit (DP350, Tiangen Biochemical Technology (Beijing) Co., Beijing, China) according to the manufacturer’s protocol.

### 2.3. Construction of CsPPO–pPIC3.5K and Codon-Optimized CsPPO–pPIC3.5K

The full-length wild-type *CsPPO* gene was amplified using the genomic DNA of Yihongzao as a template. The codon-optimized *CsPPO* was synthesized by GENEWIZ (Suzhou, China). These two fragments were then cloned into the pPIC3.5K expression vector to yield the recombinant plasmid wild-type *CsPPO–pPIC3.5K* and codon-optimized *CsPPO–pPIC3.5K*, respectively. The primers used for construction are listed in Table 1.

### 2.4. Heterologous Expression in P. pastoris

*P. pastoris* KM71 competent cells transformed with CsPPO were cultured on MD plates. A total of 30–50 larger single colonies were selected and transferred to G418-resistant YPD plates with three gradient concentrations of 0.05%, 0.1%, and 0.2%. After incubation for 1 to 2 days at 30 °C, five to ten larger strains were picked from the higher concentration of resistant plates and transferred into 10 mL of liquid YPD medium. The yeasts were then incubated at 30 °C for one day for preservation and shake flask screening. Next, a 5% inoculum of liquid YPD medium was inoculated into 50 mL of BMGY medium and incubated at 30 °C and 200 rpm for one day. The resulting solution was then transferred to sterilized 50 mL centrifuge tubes in the ultra-clean bench, sealed with a sealing film, and centrifuged at 5000 rpm for 5 min. The supernatant was then poured off and replaced with fresh BMMY medium in the centrifuge tubes containing the sediment. The yeasts were then transferred back to 250 mL conical flasks and supplemented with 1% methanol, which was replenished every 24 h. The flasks were incubated at 30 °C for 3–4 days. The enzyme activity and protein concentration of the yeast’s fluid were measured, and transformants with a high copy number were selected.

### 2.5. Extraction and Purification of Recombinant CsPPO

The extraction of yeast protein was conducted in accordance with the instructions provided by the manufacturer (Sangon Biotech, Shanghai, China) using a yeast protein extraction kit. The lysate was precipitated with 60% saturation of (NH_4_)_2_SO_4_, and then the sediment was dissolved by buffer solution. The dissolved enzyme solution was clarified by ultrafiltration using a 10 kDa Millipore ultrafiltration tube at 4 °C and 5000 *g* for 60 min and then supplemented with 20 mM pH 8 Tris-HCl buffer. The centrifugation process was repeated five times to remove small-molecular-weight contaminants and concentrate the target protein. Purification was conducted using an AKTA Purifier protein liquid chromatography system equipped with a HiTrap DEAE FF anion exchange column (Cytiva Co., Uppsala, Sweden, 5 mL). The flow rate was set at 1 mL/min, with the mobile phase A consisting of 20 mM pH 8 Tris-HCl buffer and the mobile phase B containing 1 M NaCl in 20 mM pH 8 Tris-HCl buffer. Gradient elution was applied over 25 column volumes, increasing from 0% B to 100% B. Further purification was achieved using the same AKTA Purifier system equipped with a Superdex 200 Increase 10/300 GL gel filtration column (Cytiva Co.). The flow rate was set at 0.5 mL/min, with the mobile phase comprising 0.15 M NaCl in 20 mM pH 7 phosphate sodium buffer. The proteins were eluted using isocratic conditions.

### 2.6. Detection of Protein Concentration

The Bradford protein concentration assay kit (purchased from Beyotime Biotechnology Co., Ltd., Shanghai, China) was used for the quantitative detection of protein concentration. In total, 5 μL of sample and 250 μL of G250 staining solution were mixed evenly in a 96-well plate. After standing for 10 min, the absorbance value at 595 nm was measured. The protein concentration of the sample was calculated based on the linear relationship between the concentration of standard protein and the absorbance value.

### 2.7. CsPPO Activity Assay

Before measuring enzyme activity, the concentration of CsPPO was adjusted to approximately 0.05 mg/mL. A solution of 100 mM sodium citrate buffer (pH 5.5) was prepared, and 0.1 g of proline and 1 g of catechol were dissolved in 100 mL of the buffer solution. A volume of 250 μL of the above reaction mixture was preheated for five min at 35 °C. Then, 50 μL of the enzyme solution was added, and the reaction was conducted at 35 °C. The activity of the enzyme was determined by monitoring the change in absorbance at 410 nm using a plate reader. A change in absorbance of 0.001 per minute was defined as one unit (U) of activity.

### 2.8. Determination of Optimal Temperature and Optimal pH

The CsPPO protein was incubated at temperatures between 20 °C and 45 °C separately and used for the enzyme activity assay. The temperature at which the enzyme activity is at its maximum should be defined as 100%. The optimum pH for CsPPO activity was measured at pH levels ranging from 4.0 to 7.5. The pH at which the enzyme activity is at its maximum should be defined as 100%.

### 2.9. Statistical Analysis

All results are presented as mean ± standard deviation (SD) of three replicates. The level of statistical significance among the means was analyzed by one-way ANOVA using SPSS (version 18.0; SPSS Inc., Chicago, IL, USA).

## 3. Results and Discussion

### 3.1. Cloning of Wild-Type CsPPO

The wild-type *CsPPO* in *C. sinensis* var. Yihongzao was cloned, and the full-length sequence of 1800 bp encoding 599 amino acid residues was obtained. According to the NCBI database, complete or partial sequences of the *CsPPO* gene for tea varieties such as ‘Xiangbolv’, ‘Longjing 43’, and ‘Anhui No.1’ have been published, ranging from 537 to 1800 bp [18]. Introns were not identified in the *CsPPO* genes of the tea plant. Through sequence alignment, the gene sequence of wild-type *CsPPO* cloned in this study is consistent with the previous report [19].

The wild-type *CsPPO* gene was inserted into the vector pPIC3.5K and expressed in *P. pastoris* KM71. Following methanol induction of the recombinant *P. pastoris*, no enzyme activity was detected in the supernatant of the fermentation broth. The reason for this is that pPIC3.5K is an intracellular expression vector. The recombinant cells were fragmented, and the PPO activity of the intracellular solution was measured as 30 U/mL. Therefore, this study achieved the active recombinant expression of wild-type CsPPO.

As previously reported [12], the expression of CsPPO in *E. coli* frequently results in the formation of inclusion bodies, possibly because *E. coli* is a prokaryotic organism with a relatively simple cellular structure that lacks the subcellular structures necessary to support protein post-translational modifications, including the ability to promote glycosylation and disulfide bond formation. To avoid the formation of inclusion bodies, *P. pastoris* was applied for the expression of CsPPO and the active enzyme was obtained. Wang et al. used *P. pastoris* GS115 and the vector pPICZA to express CsPPO from ‘Yingshuang’, resulting in an increase in enzyme activity of 14 U/mL compared to the empty vector control [20]. This outcome is similar to the results obtained in the present study. Despite wild-type CsPPO activity expression being achieved, the expression levels were not satisfactory.

### 3.2. Codon Optimization of CsPPO

**Figure 1:**
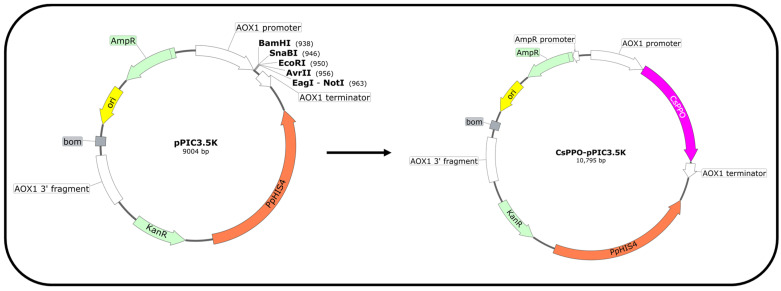
Construction of the recombinant plasmid *CsPPO–pPIC3.5K*.

As previously reported [21], heterologous genes containing many rare codons from the host are an important factor in the limitation of their expression levels. In addition, the stability of the mRNA of the target protein also affects the efficiency of protein translation. mRNA with a short half-life will be degraded by itself, resulting in a lack of template for translation, which cannot be sustained. Through sequence analysis, it was found that the wild-type *CsPPO* gene sequence contained about 14.5% of rare codons expressed in the host *P. pastoris*, including CGG (Arg), GGG (Gly), GCG (Ala), CCG (Pro), etc. It is noteworthy that the utilization frequency of these codons in *P. pastoris* was less than 10%. Based on the amino acid sequence for wild-type *CsPPO*, a codon-optimized DNA fragment of *CsPPO* was generated, and the optimized codons for the synthetic version of *CsPPO* are listed in Table 2. As shown in Table S1, the base sequence was reasonably substituted without changing the amino acid sequence of the targeted enzyme, resulting in q lower GC content and mRNA secondary structure. The codon application index (CAI) increased from 0.66 to 0.89, which was also closer to the codon bias ratio in *P. pastoris* and should improve the efficiency of codon-optimized *CsPPO* expression. Sequence analysis of the deduced codon-optimized CsPPO protein revealed a calculated molecular mass of 67.21 kDa and a predicted theoretical isoelectric point of 6.68. Subsequently, both the wild-type *CsPPO* and codon-optimized *CsPPO* genes were inserted into the expression vector pPIC3.5K and then transformed into *P. pastoris* KM71. A flow chart of recombinant plasmid construction is shown in Figure 1.

### 3.3. Activity Expression of CsPPO

**Figure 2:**
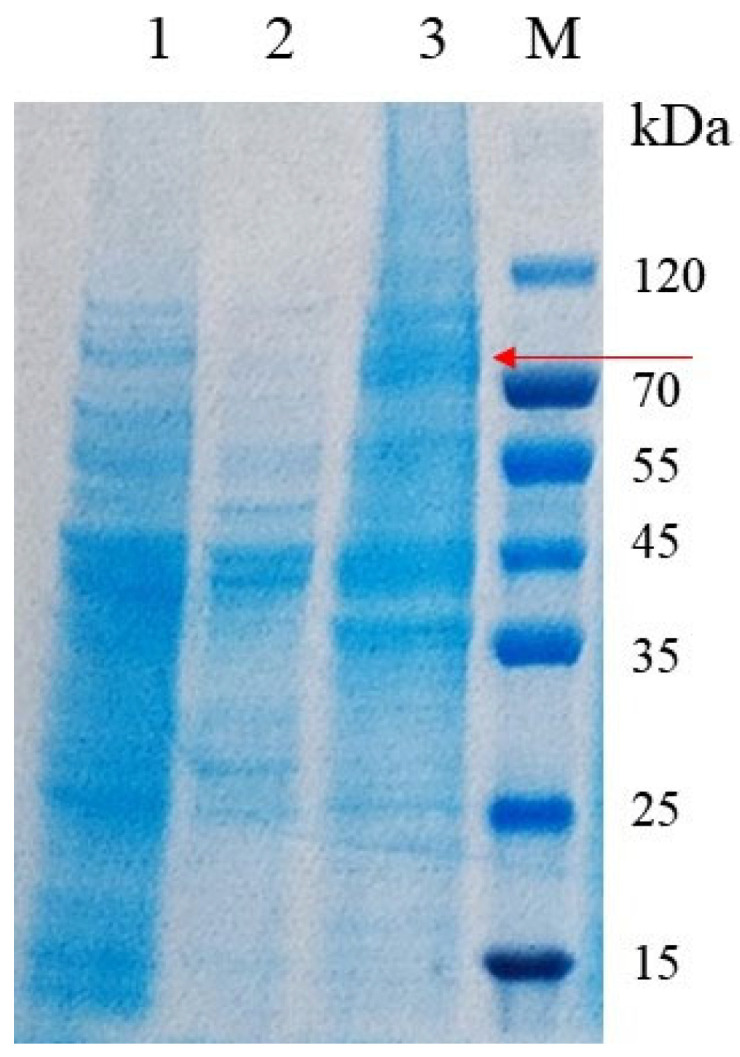
SDS-PAGE analysis of intracellular supernatant of recombinant *P. pastoris*. Lane M: protein molecular weight marker; Lane 1: the wild-type CsPPO; Lane 2: the control without CsPPO gene; Lane 3: the codon-optimized CsPPO. The red arrow represents the protein band of recombinant CsPPO.

After cultivation and methanol induction, the recombinant *P. pastoris* was collected for an analysis of enzyme production. The SDS-PAGE analysis results are shown in Figure 2. In comparison with the empty vector, a distinct protein band (the red arrow) at approximately 70 kDa was observed in both wild-type and codon-optimized CsPPO, indicating that they were successfully expressed in *P. pastoris*. Notably, codon-optimized CsPPO exhibited a more pronounced band, indicating a higher expression level compared to the wild-type. The enzyme activity of wild-type CsPPO and the total protein concentration were measured at 30 U/mL and 4.04 mg/mL, respectively. After codon optimization, the enzyme activity of CsPPO and the total protein concentration reached 1286.67 U/mL and 7.16 mg/mL, respectively. This represents a remarkable enhancement, with the enzyme activity increasing by 42.89-fold and the total protein concentration by 1.77-fold compared to those of the wild type. The remarkable increase in enzyme activity (42.89-fold) is likely attributed to enhanced mRNA stability and improved ribosome binding efficiency resulting from optimized codon usage, as reflected in the Codon Adaptation Index (CAI) increase from 0.66 to 0.89.

In a previous study [19] on the expression of Yihongzao CsPPO in a prokaryotic system using pET32a, the induced protein was observed to be larger than expected, with a molecular weight of 97 kDa. The specific enzyme activities of the soluble fraction and inclusion bodies were 5.351 × 10^4^ and 4.333 × 10^2^ U/mg, respectively. A further attempt was made to express CsPPO in *E. coli* by constructing synthetically codon-optimized *CsPPO* [22]. However, as with previous prokaryotic expression, this approach also led to the formation of CsPPO only in inclusion bodies. Despite this challenge, they explored a range of buffer compositions and refolding techniques and successfully isolated active CsPPO from the solubilized inclusion bodies. Ultimately, the refolded CsPPO exhibited an optimum pH of 5.0 and demonstrated a Vmax of 163.9 U/mg of protein when utilizing catechol as a substrate.

To obtain more active enzymes, the eukaryotic expression system was applied for the recombinant expression of CsPPO. The CsPPO-encoding gene was transferred into *P. pastoris* for expression [20], resulting in a 70 kDa active protein with an enzyme activity of 63.6 U/mL, which was 1.28 times higher than the control group (empty vector). The CsPPO activities expressed in recombinants of *P. pastoris* GS115 with secretory (pPICZA) or non-secretory (pPICZαA) vectors were 29.12 and 26.92 U/mg, respectively, while the enzyme activities of the control groups with empty vectors of pPICZA and pPICZαA were 25.02 and 21.81 U/mg, respectively [16]. These studies attempted to express the active wild-type PPO protein in the yeast strain GS115. However, the expression level of the wild-type PPO protein only showed 1.16- to 1.28-fold increases compared to the corresponding control groups. In this study, we expressed both the wild-type and codon-optimized PPO proteins in strain KM71. The activity of the codon-optimized PPO protein increased significantly, with its enzymatic activity being more than 40 times higher than that of the wild-type protein, underscoring the critical role of codon adaptation in eukaryotic systems. This study achieved the active expression of CsPPO through codon optimization, and the enzyme activity was significantly improved compared to the previous results.

### 3.4. Property Analysis of Codon-Optimized CsPPO

**Figure 3:**
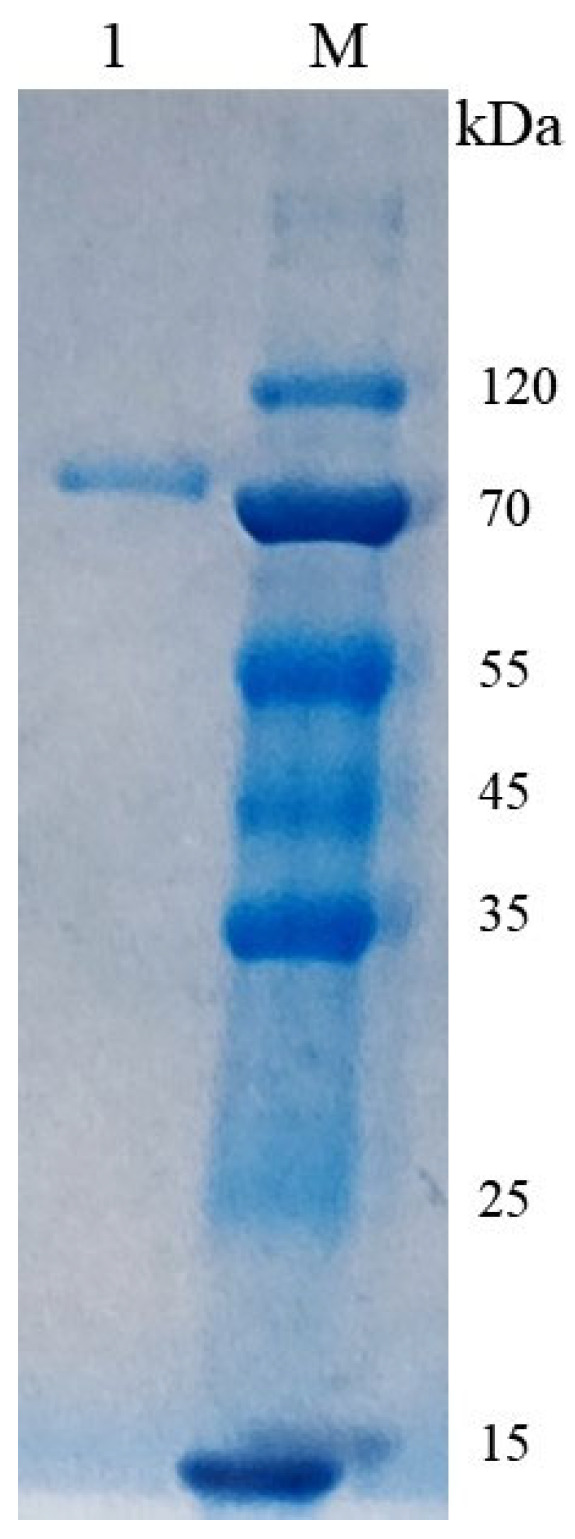
SDS-PAGE analysis of the purified CsPPO. Lane M: protein molecular weight marker; Lane 1: purified CsPPO.

The crude enzyme solution was purified by ammonium sulfate precipitation, ultrafiltration, DEAE anion exchange chromatography, and Superdex-200 gel filtration chromatography. The purified CsPPO exhibited a single band at around 70 kDa (Figure 3). As shown in Table S2, the specific enzyme activity of purified CsPPO was 8315.10 U/mg, which was 46.27 times that of the crude enzyme. The yield of CsPPO at Superdex-200 step was 2.46%.

**Figure 4:**
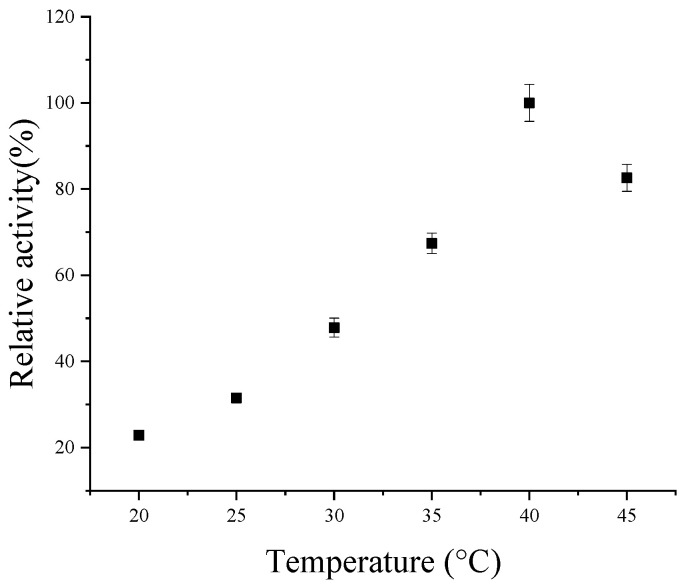
Effects of temperature on codon-optimized CsPPO activity.

The effect of temperature on the activities of codon-optimized CsPPO is illustrated in Figure 4 and Table S3. At 20 °C, the activity was only 22% of the maximum; at 25 °C, there was an increase to 25%; at 30 °C, there was a significant rise to 43%; and at 35 °C, there was a further increase to 63%. It is notable that, at 40 °C, the enzyme activity peaked at 100% of the maximum activity, which was considered the optimal condition for enzyme activity. However, when the temperature continued to rise to 45 °C, the enzyme activity declined to approximately 81%.

The optimal temperature range for CsPPO from different tea leaves was mostly observed to be between 25 and 50 °C. Zhao et al. found that the optimal temperature for CsPPO isolated from fresh tea leaves (*C. sinensis* cv. Zhuyeqi) was 50 °C [23]. Liu et al. compared the enzymatic properties of soluble and membrane-bound CsPPO from *C. sinensis* cv. ‘Longjing 43’, finding that their optimal temperatures were 25 °C and 30 °C, respectively [24]. In a further study, two CsPPOs were isolated and purified from *C. sinensis* var. ‘Zhenghedabai’, and their optimal temperatures were determined to be 33 °C and 38 °C, respectively [25]. Therefore, the optimal temperature of codon-optimized CsPPO in this study falls within the range reported in the previous studies.

**Figure 5:**
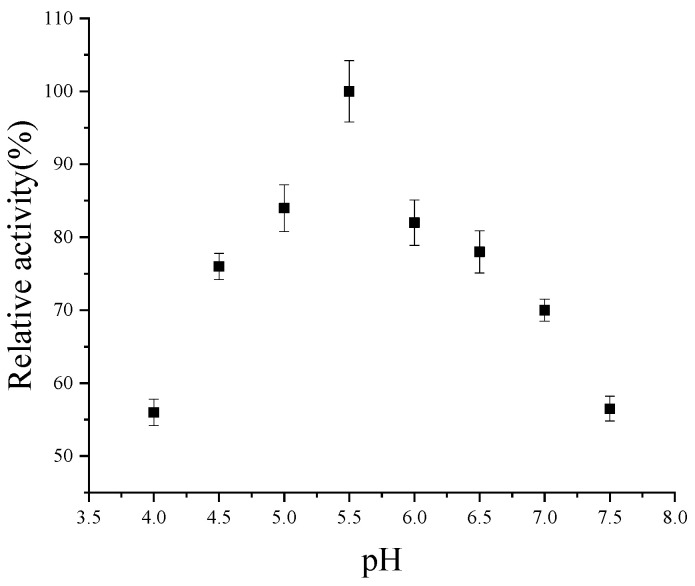
Effects of pH on codon-optimized CsPPO activity.

As shown in Figure 5 and Table S4, the highest level of codon-optimized CsPPO activity was observed at pH 5.5, while the activity significantly decreased when the pH deviated from the optimum pH. At pHs of 4.0, 4.5, and 5.0, the codon-optimized CsPPO activity was reduced to 56%, 75%, and 83% of its peak performance, respectively. Similarly, an increase in pH resulted in a decline in PPO activity, with 81%, 76%, 70%, and 55% of the maximum activity observed at pH 6.0, 6.5, and 7.0, respectively.

According to reports, the optimal pH range for CsPPO derived from different tea plants is 5.0–6.2. CsPPO, with a molecular weight of 72 kDa, was purified from an Indian tea leaf, and its optimal pH was determined to be 5.0 [26]. Thermo-tolerant CsPPO was isolated from a black tea infusion, with an optimal pH of 6.2 [27]. The optimal pH values for two types of CsPPO from Huangjinya tea were 6.0 and 5.5, respectively [28], with one of them having the same optimal pH as the CsPPO used in this study.

## 4. Conclusions

In this study, we successfully cloned the wild-type CsPPO gene (1800 bp) and expressed it in *P. pastoris* KM71. By replacing rare codons with synonymous ones, we achieved a significant enhancement in both enzyme activity and protein concentration. Specifically, the enzyme activity of codon-optimized CsPPO reached 1286.67 U/mL, representing 42.89-fold increases over that of the wild type. The optimal temperature and pH for codon-optimized CsPPO were 40 °C and 5.5, respectively, consistent with previous reports. These results demonstrate the effectiveness of our codon optimization strategy and highlight the efficient expression and activity of CsPPO in a eukaryotic expression system. This work provides a foundation for the large-scale production and widespread application of CsPPO.

## Figures and Tables

**Table 1:** Primers used for construction of wild-type *CsPPO–pPIC3.5K* and codon-optimized *CsPPO–pPIC3.5K* (*CO-CsPPO–pPIC3.5K*).

Primers	Primer Sequence (5′–3′)
*WT-CsPPO-F*	aactaattattcgaaggatccATGGCTTCCATTCTCCCTCC
*WT-CsPPO-R*	cgcggccgccctagggaattcTTAAGAATCAAACTCAATCTTGACACC
*CO-CsPPO-F*	aactaattattcgaaggatccATGGCTTCCATCTTGCCACC
*CO-CsPPO-R*	cgcggccgccctagggaattcTTAGGAGTCGAACTCAATCTTGACACC

**Table 2:** Comparisons of *CsPPO* gene sequence before (R) and after (P) codon optimization.

**R 1-60**	ATG GCT TCC ATT CTC CCT CCA ACC ACC ACC AAG ACT ACC ACC ACC TCC TCC ACC CTC TAT ATG GCT TCC ATC TTG CCA CCA ACT ACT ACC AAG ACT ACT ACT ACC TCC TCC ACC TTG TAC
**P 1-60**	
**R 61-120**	TCT TAT CCC ATT TTC AAG AAC ACC TCT AAA ATT CCC ACA ATT AGA AAG CAT AAC CAT TCC TCT TAC CCA ATC TTC AAG AAC ACC TCC AAG ATT CCA ACC ATC AGA AAG CAC AAC CAT TCC
**P 61-120**	
**R 121-180**	TTC AAT GTG TCA TGC AGC AAA GCC AAA GAT AGT GAC CCA AAC CTT ACT CCC CCC TCC CAA TTC AAC GTG TCC TGT TCC AAG GCT AAG GAT TCT GAC CCA AAC TTG ACT CCA CCA TCT CAG
**P 121-180**	
**R 181-240**	AAT ACA CAA ACC TCC CTA GGG AAG TTT GAT AGG AGA AAC ATG GTC ATT GGC TTA GGA GGC AAC ACT CAA ACT TCC CTG GGA AAG TTC GAC AGA CGT AAC ATG GTT ATT GGT CTT GGT GGT
**P 181-240**	
**R 241-300**	CTC TAC GGA GCA GCC GGA CTC ACC GAC ACT GAC CCA CCA GCC TTG GCC GCT CCG GTC ACC CTG TAC GGT GCT GCT GGT TTG ACT GAT ACT GAT CCA CCA GCT TTG GCT GCT CCA GTT ACT
**P 241-300**	
**R 301-360**	GCC CCG GAT TTA TCC AAA TGC GGG GCG GCG GAT TTA CCC GCG GAT GCA AAA CCG ACC AAC GCT CCA GAT TTG TCT AAG TGT GGT GCT GCA GAT TTG CCA GCT GAT GCT AAG CCA ACT AAC
**P 301-360**	
**R 361-420**	TGT TGT CCT CCA AAA ACC AAC AAA ATC ATC GAA TTC AAG CTC CCT CCT CCC TCC AAC ATT TGT TGT CCA CCA AAG ACT AAC AAG ATC ATC GAG TTC AAG CTG CCA CCA CCA TCC AAC ATC
**P 361-420**	
**R 421-480**	TTA CGA GTC CGA CCC GCG GCC CAT TTA GCC GAC GAA AAA TAC ATA GCC AAG TTT TCT AAA TTG AGA GTT AGA CCA GCT GCT CAC TTG GCT GAC GAG AAG TAC ATT GCT AAG TTC TCC AAG
**P 421-480**	
**R 481-540**	GCC CTC CAA CTC ATG AAA TCG CTC CCC GAT GAC GAC CCA CGA AGC TTC AAG CAA CAA TCC GCT CTG CAG CTG ATG AAG TCT TTG CCT GAT GAT GAC CCA AGA TCC TTC AAG CAG CAG TCC
**P 481-540**	
**R 541-600**	AAT ATT CAC TGC GCT TAT TGC GAA GGC GCT TAT CAC CAA GTC GGT TTT CCA AGC ACA GAA AAC ATT CAC TGT GCT TAC TGT GAA GGT GCC TAC CAC CAA GTT GGT TTC CCA TCT ACT GAG
**P 541-600**	
**R 601-660**	CTC CAA GTT CAC AAC TCA TGG CTC TTC TTT CCC TTC CAT AGA TTC TAT CTC TAC TTC TTC TTG CAG GTT CAC AAC TCC TGG CTG TTT TTC CCA TTC CAC AGA TTC TAC CTG TAC TTC TTC
**P 601-660**	
**R 661-720**	GAA AAG ATT TTG GGA ATG CTG CTC GAT GAT CCA GCG TTT GCA ATT CCT TTT TGG AAT TGG GAG AAG ATC CTG GGT ATG TTG TTG GAC GAT CCA GCT TTC GCT ATC CCA TTC TGG AAC TGG
**P 661-720**	
**R 721-780**	GAT TCT CCG GCG GGC ATG AAA ATA CCC GCC ATG TAT GCG GAC ATA AAT TCG CCA CTC TAT GAT TCT CCA GCT GGT ATG AAG ATC CCA GCT ATG TAC GCT GAC ATT AAC TCC CCA CTG TAC
**P 721-780**	
**R 781-840**	AAC CGT CTC CGT GAC GCC AAA CAC CAG CCA CCG ACA TTA ATT GAC CTT GAC TAC AAT TTG AAC AGA TTG AGA GAT GCC AAG CAC CAG CCA CCA ACC TTG ATT GAT TTG GAC TAC AAC CTG
**P 781-840**	
**R 841-900**	ACT GAT CCG AAA AAT GTC GAT GAG GAG AAG CAG AAG TTG AGA AAT CTA ACT ATA ATG TAC ACT GAC CCA AAG AAC GTT GAC GAA GAG AAG CAG AAG TTG AGG AAC CTG ACC ATC ATG TAC
**P 841-900**	
**R 901-960**	CGA CAA GTG GTG TCG GGT GGG AAA ACG CCT CGG CTT TTC CTT GGA AGC TCG TAC CGT GCG AGA CAG GTT GTT TCC GGT GGT AAG ACC CCT AGA CTG TTC TTG GGT TCT TCT TAC AGA GCT
**P 901-960**	
**R 961-1020**	GGA GAT GAC CCG GAC CCA GGT GCC GGG TCC CTG GAG AAC ATC CCG CAT GGT CCG GTT CAC GGT GAT GAC CCT GAT CCA GGT GCT GGT TCT TTG GAA AAC ATT CCA CAT GGT CCA GTC CAC
**P 961-1020**	
**R 1021-1080**	ATA TGG TGC GGG GAC CGC ACC CAG CCG AAT CTA GAA GAC ATG GGG AAC TTC TAC TCT GCG ATC TGG TGT GGT GAT AGA ACT CAG CCA AAC TTG GAG GAC ATG GGT AAC TTC TAC TCC GCT
**P 1021-1080**	
**R 1081-1140**	GGA CGA GAT CCG ATC TTC TAC GGT CAT CAC GCG AAC GTC GAT CGG ATC TGG ACG GTG TGG GGT AGA GAT CCA ATC TTC TAC GGT CAT CAC GCC AAC GTT GAC AGA ATC TGG ACT GTC TGG
**P 1081-1140**	
**R 1141-1200**	AAG ACA TTA GGA GGA AAA CGA AAC GAT TTC AAG GAT TCG GAT TGT TTG AAT TCA GAG TTC AAA ACC CTT GGT GGA AAG AGA AAC GAC TTC AAG GAC TCC GAC TGT CTG AAC TCC GAG TTT
**P 1141-1200**	
**R 1201-1260**	ACC TTT TAC GAC GAA AAT GCT CAG CTT GTG ACT GTA AAA GTA AAA GAG AGT TTG GAT CAT ACT TTC TAC GAC GAG AAC GCC CAG TTG GTT ACC GTT AAG GTC AAA GAA TCC CTG GAC CAC
**P 1201-1260**	
**R 1261-1320**	CGA AAA CTC GGC TAC GTC TAC CAA GAC GTG GAA ATT CCA TGG CTA AAC GCT CGA CCC AGT AGA AAG CTG GGT TAC GTT TAC CAG GAC GTT GAG ATC CCA TGG TTG AAC GCT AGA CCA TCT
**P 1261-1320**	
**R 1321-1380**	CCT CGT ATT TCA AAT TTT TTT CGA AAA ATA AAG AAC AAG GCC GGG ATA GCA ATG GCG ACA CCA AGA ATC TCC AAC TTC TTC AGA AAG ATC AAG AAC AAG GCC GGT ATC GCT ATG GCT ACT
**P 1321-1380**	
**R 1381-1440**	GAG ACA CTG GAT TCT GCT GCC ATT GTA TTC CCA AGA AAG CTT GAT GAG GTG GTG AAG GTG GAA ACT TTG GAT TCC GCC GCT ATC GTT TTC CCA AGA AAG TTG GAT GAG GTC GTC AAG GTT
**P 1381-1440**	
**R 1441-1500**	GTG GTG AAG CGG CCG ACG AAG TCG AGG AGT GAG AGA GAG AAG GAA GAA GAG GAG GAG GTG GTT GTC AAG AGG CCA ACT AAG TCC AGA TCC GAG AGA GAG AAA GAG GAA GAA GAA GAG GTT
**P 1441-1500**	
**R 1501-1560**	GTG GTA GTG GAG GGG ATA GAG ATG GAG AGA GAT GTG TCT GTG AAG TTT GAT GTG TTT ATT GTC GTC GTT GAG GGT ATC GAA ATG GAA AGG GAC GTT TCC GTT AAG TTC GAC GTG TTC ATT
**P 1501-1560**	
**R 1561-1620**	AAC GAC GAA GAC GAG GCG GCA AGT GGG CCG GAG AAG ACA GAG TTC GCC GGA AGC TTT GTG AAC GAC GAG GAC GAA GCT GCT TCT GGT CCA GAA AAG ACT GAA TTT GCC GGT TCC TTC GTG
**P 1561-1620**	
**R 1621-1680**	AAT GTG CCG CGT AAA CAT AAG CAT GAC AAG AAG ATA AGG ACT AGT TTG AGG TTG GGG ATA AAC GTG CCA AGA AAA CAT AAG CAC GAC AAG AAG ATC AGG ACC TCC TTG AGA TTG GGT ATC
**P 1621-1680**	
**R 1681-1740**	ACT GAG CTA TTG GAG GAC TTG GAA GCT GAA GAT GAT GAG AGC GTG CTG GTG ACT TTG GTC ACC GAG TTG TTG GAG GAC TTG GAA GCT GAA GAT GAC GAG TCC GTT TTG GTC ACT TTG GTC
**P 1681-1740**	
**R 1741-1800**	CCT AGA TAT GGG TCT GAT GCT GTC ACT ATT GGT GGT GTC AAG ATT GAG TTT GAT TCT TAA CCA AGA TAC GGT TCC GAC GCT GTT ACT ATT GGT GGT GTC AAG ATT GAG TTC GAC TCC TAA
**P 1741-1800**	

Note: The optimized codons are highlighted in green.

## Data Availability

The original contributions presented in the study are included in the article/Supplementary Material, further inquiries can be directed to the corresponding author.

## Supplementary Materials

The following supporting information can be downloaded at: https://www.mdpi.com/article/10.3390/foods14152749/s1, Table S1: The comparison of key parameters between the wild-type *CsPPO* gene and the codon-optimized *CsPPO* gene; Table S2: Summary of the isolation and purification of codon-optimized CsPPO; Table S3: Effects of temperature on codon-optimized CsPPO activity; Table S4: Effects of pH on codon-optimized CsPPO activity.
