# Supplementary material for: Activity Expression and Property Analysis of Codon-Optimized Polyphenol Oxidase from Camellia sinensis in Pichia pastoris KM71
Source: Foods. 2025 Aug 6;14(15):2749. doi: 10.3390/foods14152749 (PMC12345873; doi:10.3390/foods14152749)
Supplement: Supplementary file 1 [file foods-14-02749-s001.zip › foods-3758603-supplementary.pdf]

Table S1. The comparison of key parameters between the wild-type *CsPPO* gene and the codon-optimized *CsPPO* gene.

Gene	CAI [a]	GC (%)	ΔG (kcal/mol) [b]	Change rate of codon (%)
wild-type gene	0.64	48.89	-564.90	
codon-optimized gene	0.87	47.83	-514.10	62.33

Note: [a] Represents codon application index; the positive correlation is shown between the CAI and gene expression level.

[b] Represents the minimum of free energy of mRNA folding.

Table S2. Summary of the isolation and purification of codon optimized CsPPO.

Purification step	Total activity (U)	Total protein (mg)	Specific activity (U/mg)	Yield (%)	Purification fold
Crude enzyme	102933.60	572.80	179.70	100.00	1.00
$(NH_4)_2SO_4$ precipitate and ultrafiltration	45333.33	29.67	1528.02	44.04	8.50
DEAE-Sepharose	8290.00	2.36	3515.89	8.05	19.57
Superdex-200	2533.33	0.30	8315.10	2.46	46.27

Table S3. Effects of temperature on codon optimized CsPPO activity.

Temperature (°C)	Enzyme activity (U/mL)
20	859.05 ± 42.95
25	1186.30 ± 40.50
30	1799.91 ± 82.47
35	2536.23 ± 89.18
40	3763.44 ± 161.83
45	3108.93 ± 117.81

Table S4. Effects of pH on codon optimized CsPPO activity.

pH	Enzyme activity (U/mL)
4	1417.03 ± 45.55
4.5	1923.11 ± 45.55
5	2125.54 ± 80.97
5.5	2530.41 ± 106.28
6	2074.94 ± 78.44
6.5	1973.72 ± 73.38
7	1771.29 ± 37.96
7.5	1429.68 ± 43.02
